# The Effect of Vitamin D Supplementation on Incidence of Type 2 Diabetes: A Systematic Review

**DOI:** 10.7759/cureus.36775

**Published:** 2023-03-27

**Authors:** Zahid Khan, Syed Aun Muhammad, Jonard Carpio, Yousif Yousif, Amresh Gul, Sahar Hamid, Animesh Gupta

**Affiliations:** 1 Acute Medicine, Mid and South Essex NHS Foundation Trust, Southend on Sea, GBR; 2 Cardiology, Bart’s Heart Centre, London, GBR; 3 Cardiology and General Medicine, Barking, Havering and Redbridge University Hospitals NHS Trust, London, GBR; 4 Cardiology, Royal Free Hospital, London, GBR; 5 Cardiology, Mid and South Essex NHS Foundation Trust, Southend on Sea, GBR; 6 Internal Medicine, Mid and South Essex NHS Foundation Trust, Southend on Sea, GBR; 7 Internal Medicine, Barking, Havering and Redbridge University Hospitals NHS Trust, London, GBR; 8 General Practice, Lifeline Hospital, Salalah, OMN; 9 Acute Internal Medicine, Southend University Hospital NHS Trust, Southend on Sea, GBR; 10 Acute Internal Medicine/Intensive care, Barking, Havering and Redbridge Hospital NHS Trust, London, GBR

**Keywords:** preventative medicine, colecalciferol and type 2 diabetes, vitamin d and diabetes, prevention of diabetes, duration of diabetes mellitus, risk of cardiovascular diseases, systematic review and meta analysis, vitamin-d deficiency, vitamin d level, diabetes mellitus type 2

## Abstract

With the clinical increase in Type 2 Diabetes worldwide, several interventions to decrease its incidence have been investigated. One such intervention is Vitamin D supplementation, as it affects Insulin secretion from the pancreas and Insulin receptors in the cells of the body. This systematic review addresses whether or not Vitamin D supplementation has a role in reducing the risk of developing Type 2 Diabetes. Systematic searches were conducted on PubMed, and Cochrane Library mainly but also checked Google Scholar. Randomized controlled trials, systematic trials and cohort studies were retrieved that included keywords pertaining to Vitamin D supplementation and the incidence of Type 2 Diabetes. Exclusion criteria included studies that looked at different forms of Diabetes, studies including patients aged less than 18 or more than 85 years of age and studies that were not English language. For all the trials identified, the incidence of Type 2 Diabetes among the cohort receiving vitamin D supplementation was compared to the cohort receiving placebo medication. Additionally, the Homeostatic Model Assessment for Insulin Resistance (HOMA-IR) was analyzed to observe if there was a difference between Insulin resistance among these two cohorts between the start of the trials and the end. Thirteen randomized controlled trials were identified. Seven of these identified incidences of Type 2 Diabetes as a research outcome, out of which six showed no statistically significant impact of vitamin D on the incidence of Type 2 Diabetes. Out of the 13 trials, 10 analyzed the impact of vitamin D supplementation on patients’ HOMA-IR. In six of these trials, patients receiving vitamin D supplementation had a decrease in their HOMA-IR, while it increased in 4 trials. In seven of the ten trials that analyzed for HOMA-IR, the HOMA-IR was less in the vitamin D cohort than the placebo cohort. There is insufficient evidence to suggest that vitamin D supplementation significantly reduces the incidence of Type 2 Diabetes despite its effects on insulin resistance. Further research in this area would be helpful in order to influence clinical guidelines on vitamin D supplementation among patients at risk of Type 2 Diabetes.

## Introduction and background

Type-2 Diabetes Mellitus (T2DM) prevalence is significant worldwide and the economic burden of the disease is staggering. In the UK, it is estimated that of the 4.7 million who have a diagnosis of diabetes, 90% have T2DM. This has more than doubled in the past 20 years and it is predicted that this number will exceed 5.5 million in 2030 [1]. In 2010/11, the NHS (National Health Service, UK) spent £8.8bn in treating T2DM and its complications and it is estimated that these costs will nearly double in 2035 [2]. Furthermore, the disease and its complications put a significant burden on those affected despite existing beneficial pharmacological therapies. Therefore, many studies have aimed at evaluating preventive measures against the disease [3] resulting in emerging cost-effective therapies being postulated in the hopes of further controlling blood glucose levels and consequently reducing the risk of developing T2DM.

**Figure 1:**
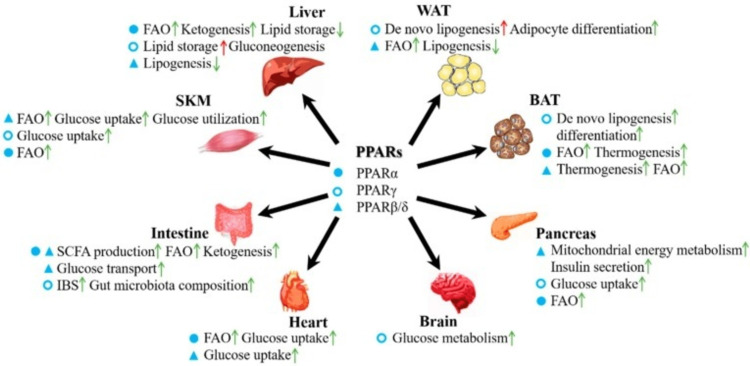
Roles of PPARs in the energy metabolism of various organs PPAR - Peroxisome proliferator-activated receptors [9] Permission has been obtained from the author/journal for reproducing this figure.

**Figure 2:**
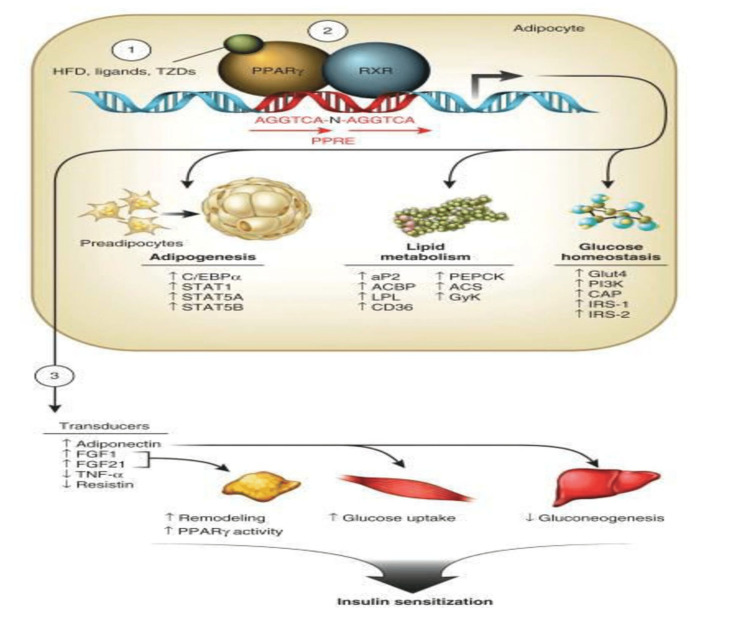
Mechanism of action of peroxisome proliferator-activated response on tissues via DNA in a cellular level Permission has been obtained from the author/journal for reproducing this figure [10].

One particular therapy that has been extensively investigated is Vitamin D. Famously known for its role in calcium metabolism, vitamin D is converted to 25(OH)D in the liver and then in the kidneys to form 1,25-dihydroxycholecalciferol or 1,25(OH)2D, the active metabolite that is responsible for intestinal absorption of calcium and bone mineralization. Therefore, it is well established that severe deficiency of the vitamin results in poor bone development defined as osteomalacia (in adults) and rickets (in children). However, vitamin D is less widely recognized for its role in improving pancreatic beta cell function and insulin sensitivity. Its mechanism is mainly via the promotion of the expression of insulin receptors and enabling peroxisome proliferator-activated response-δ (Figure 1) [4], which promotes glucose uptake in peripheral tissues, similar to the anti-diabetic drug pioglitazones mechanism of action (Figure 2). It also stimulates insulin secretion via vitamin D receptor activation in the beta cell of the pancreas [5]. Therefore, vitamin D has been found to improve fasting glucose, HbA1c level in patients [6,7] and the risk of developing T2DM. The same can be said for the opposite. The lack thereof could increase the risk of developing T2DM due to decreased glucose uptake and insulin secretion. This was evidenced by a meta-analysis which found that there was a 54% likelihood of pre-diabetes in those with vitamin D deficiency [7]. This is furthered by a cross-sectional study in Indian women, in which vitamin D insufficiency was found to be widespread among those with T2DM [8]. With this, including being readily available and inexpensive as oral formulations, recommending vitamin D to reduce the risk of developing T2DM is promising and hangs in the balance and can only be advised based on appropriate evidence. Hence, this study aims to review the recent evidence of the association between vitamin D status and the risk of developing T2DM and the effect of vitamin D in those studies.

## Review

Materials and Methods

Search Strategy

The study is registered with PROSPERO and the National Institute of health research under registration number CRD42022363865. A systematic review was conducted utilizing multiple search engines in order to establish research performed on the association between Vitamin D deficiency and the development of Type 2 Diabetes. The external databases used were Cochrane Library and PubMed. Key terms used for the search were “Type 2 diabetes,” “Vitamin D,” “Lack of Vitamin D,” and “Vitamin D and Diabetes.” Studies with “type 1 and type 2 diabetes” in the title were excluded. A total of 6,789 articles were identified.

Criteria for Selection

**Figure 3:**
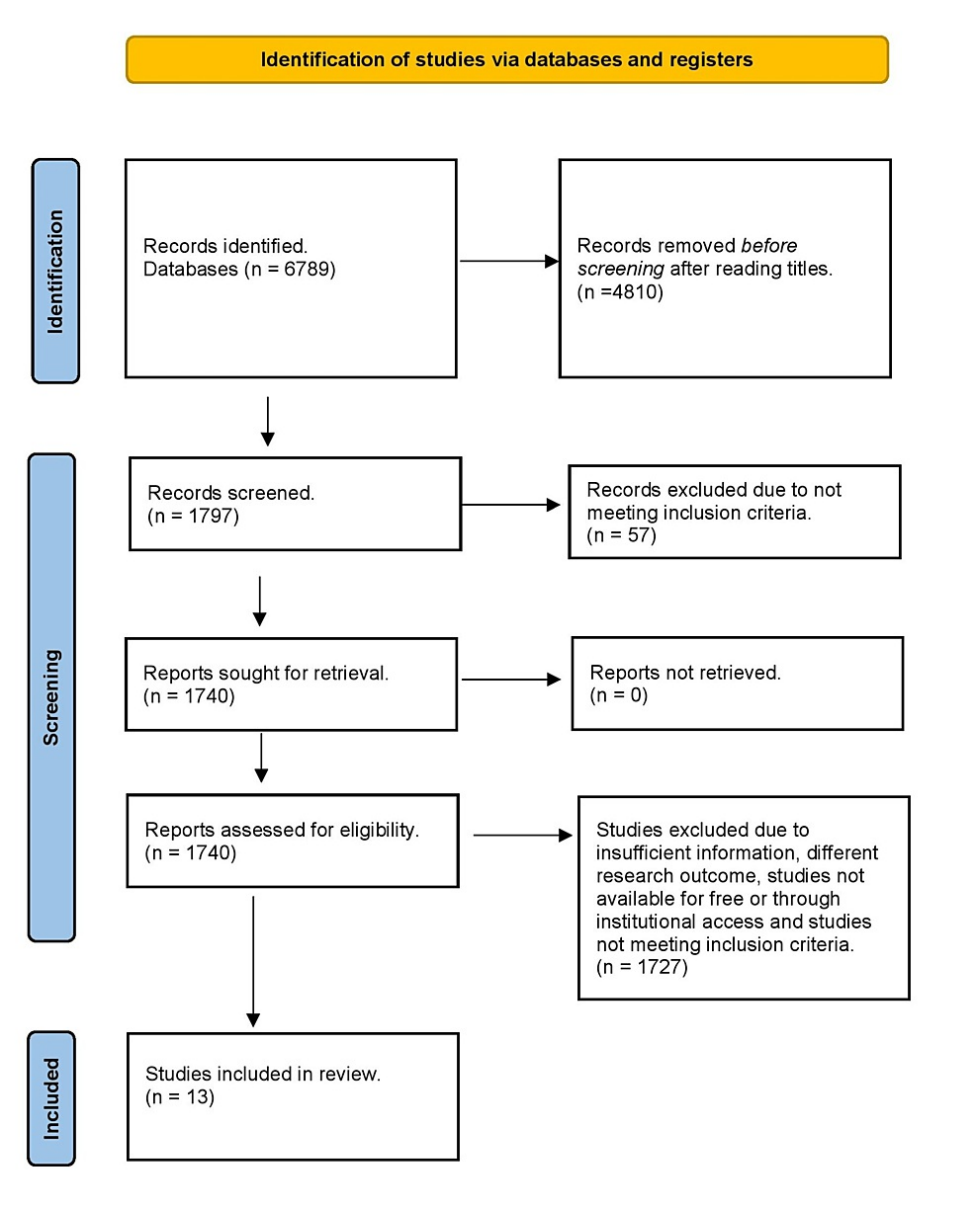
Preferred Reporting Items for Systematic Reviews and Meta-Analyses (PRISMA) chart.

Studies were required to be either randomized controlled trials or observational studies such as cohort studies. The question of interest was specifically whether or not having a deficiency of Vitamin D resulted in an increased risk of developing Type 2 Diabetes, rather than whether or not treatment of Vitamin D deficiency improved pre-existing Type 2 Diabetes. The onset of other forms of Diabetes, such as Type 1 Diabetes, Modified Onset Diabetes of the Young or Latent Autoimmune Diabetes in Adults were not included in this study. Additionally, articles that talked about Diabetes Insipidus were also excluded. Exclusion criteria included patients less than 18 years of age or patients more than 85 years of age, studies that were not in the English language or not freely available. There were a number of reasons for why research articles were identified and not included in the final study. The vast majority of the articles (4,810) were excluded as the title included Vitamin D or Diabetes, but they were not relevant to the study due to the outcome researched being different. After screening the titles, abstracts were analyzed and excluded if their aims and objectives were not relevant. From the 1,727 studies, 1380 studies were looking at other variables and were not assessing the role of vitamin D in the prevention of diabetes, 285 studies included patients with established diabetes and 62 studies did not provide enough information for us to draw conclusion. Studies that did not go into sufficient detail or had missing data were also excluded. Some articles were not accessible and were included in the study. Lastly, articles that were not in English language were not included in the study as shown in Preferred Reporting Items for Systematic Reviews and Meta-Analyses (PRISMA) chart (Figure 3). Case reports, case series and editorials that were identified were excluded.

Results

After applying the inclusion and exclusion criteria outlined above, 13 randomised control trials were identified involving patients with pre-diabetes. Table 1 highlights the trials, including how many patients were involved in each trial, their mean age, the distribution of their sex, their ethnicity and baseline diabetic status. There was a wide range of time periods that the trials ran for, ranging from three months to five years. The dosage of vitamin D that was given to patients varied, with most of the trials using a form of Vitamin D3, either administered daily, weekly or monthly. Table 2 highlights the results of these trials, outlining the duration that each trial ran, the dose of vitamin D used, the number of patients taking vitamin D vs placebo and the incidence of diabetes, where provided. The hazard ratios are also included, where they have been provided in the papers.

**Table 1:** Patient demographics in the identified trials

Author	Total No. of patients	Mean Age (years)	Sex	Ethnicity	Baseline diabetic status
Pittas et al. [11]	2423	60	Males: 45.2% Females: 44.8%	White: 66.7% Non-white: 33.3%	Prediabetes
Dutta et al. [12]	170	46 – 48	Males: 40.4% Females: 59.6%	Non-white: 100%	Prediabetes
Jorde et al. [13]	511	62	Males: 61% Females: 39%	Not provided	Prediabetes
Salehpour et al. [14]	77	37-38	Males: 0% Females: 100%	Not provided (although based in Iran)	Obese/overweight
Al Thani et al. [15]	209	44.89 – 45.51	Males: 83.3% Females: 16.7%	Arab: 54%	Prediabetes
Moreira-Lucas et al. [16]	71	45.6-49.1	Males: 39.7% Females: 61.3%	White: 49.2%	Prediabetes (55.6%)
Oosterwerff et al. [17]	130	48.9-51.5	Males: 40% Females: 60%	Non-Western: 100% Moroccan: 38%	Prediabetes
Rasouli et al. [18]	1774	60.5	Males: 56.4% Females: 43.6%	White: 69.1% Black: 22.7% Asian: 5.5%	Prediabetes
Wallace et al. [19]	66	53.3	Males: 59% Females: 41%	Not provided	Prediabetes
Forouhi et al. [20]	340	52.4-53.5	Males: 57% Females: 43%	White: 79% Non-white: 21%	Prediabetes
Ahmed et al. [21]	120	41.1-41.6	Not provided	Not provided	Prediabetes
Kawahara et al. [22]	1256	61.3	Males: 54.5% Females 45.5%	Japanese: 100%	Prediabetes
Sollid et al. [23]	511	62.1	Males: 61.4% Females: 39.6%	Not provided	Prediabetes

**Table 2:** Results from each of the included trials outlining the incidence of diabetes

Author	Trial period	Vitamin D dose	No. of patients taking Vit D at start of study (n, %)	No. of patients taking placebo at start of study (n, %)	No. of patients taking Vit D at end of study (n, %)	No. of patients taking placebo at end of study (n, %)	Onset of Diabetes among Vit D Cohort at end of study (n, %)	Onset of Diabetes among placebo cohort at end of study (n, %)	Hazard Ratio of Vit D compared to Placebo
Pittas et al. [11]	48 months	Vit D3 4000 IU/day	1211, 50%	1212, 50%	1201, 50%	1199, 50%	293, 24.4%	323, 26.9%	0.88
Dutta et al. [12]	28 months	Vit D3 60,000 IU/week for 8 weeks then monthly - Calcium supplemented in all groups	68 54.4%	57, 45.6%	55, 52.8%	49, 47.2%	6, 10.9%	13, 26.5%	Not given
Jorde et al. [13]	60 months	Vit D3 20,000 IU/week	256, 50%	255, 50%	206, 49.5%	210, 51.5%	103, 50%	112, 53.3%	0.9
Salehpour et al. [14]	12 weeks	Vit D3 1000 IU/day	42, 49.4%	43, 50.6%	39, 50.6%	38, 49.4%	Not provided, although no difference between cohort and control group	Not provided, although no difference between cohort and control group	Not provided
Al Thani et al. [15]	6 months	Vit D3 30,000 IU/week	110, 52.6%	99, 47.3%	57, 43.2%	75, 56.8%	Not provided, although no difference between cohort and control group	Not provided, although no difference between cohort and control group	Not given
Moreira-Lucas et al. [16]	24 weeks	Vit D3 28,000 IU/week via cheese	35, 49.2%	36, 51.8%	32, 50.8%	31, 49.2%	Not provided, although no difference between cohort and control group	Not provided, although no difference between cohort and control group	Not given
Oosterwerff et al. [17]	16 weeks	Vit D3 1200 IU/day (Calcium supplemented in both groups)	65, 50%	65, 50%	53, 48.2%	57, 51.8%	4, 7.5%	8, 14%	OR 0.89
Rasouli et al. [18]	24 months	Vit D3 4000 IU/day	1211, 50%	1212, 50%	888, 50%	886, 50%	116, 13.1%	159 17.9%	0.7
Wallace et al. [19]	26 weeks	Vit D3 3000 IU/day	35, 53%	31, 47%	34, 53.1%	30, 46.9%	Not provided, although no difference between cohort and control group	Not provided, although no difference between cohort and control group	Not provided
Forouhi et al. [20]	4 months	Vit D3 100,000 IU/month	112, 33%	114, 33.5%	99, 34.7%	92, 32.3%	Not provided, although no difference between cohort and control group	Not provided, although no difference between cohort and control group	Not provided
		Vit D2 100,000 IU/month	114, 33.5%		94, 33%		Not provided, although no difference between cohort and control group		
Ahmed et al. [21]	12 weeks	Vit D3 60,000 IU/week	60, 50%	60, 50%	52, 51.4%	49, 48.6%	Not provided, although no difference between cohort and control group	Not provided, although no difference between cohort and control group	Not provided
Kawahara et al. [22]	36 months	Eldecalcitol 30 IU/day	630, 50.1%	626, 49.9%	556, 50%	555, 50%	79, 14.2%	89, 16%	0.87
Sollid et al. [23]	12 months	Vit D3 20,000 IU/week	256, 50%	255, 50%	242, 50%	242, 50%	39, 16.1%	41, 16.9%	Not provided

**Figure 4:**
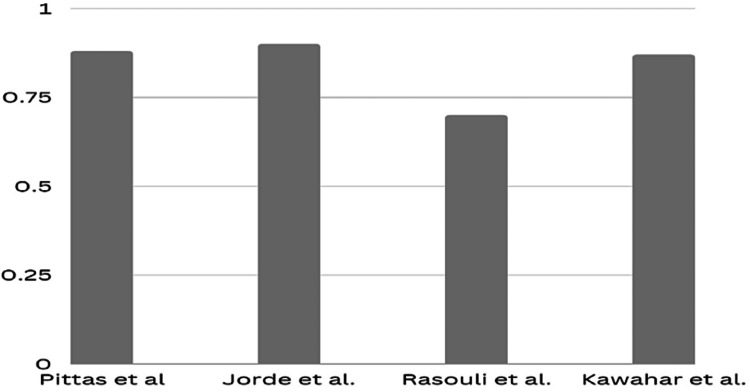
Bar graph outlining the hazard ratios of the incidence of diabetes among patients taking Vitamin D vs patients taking placebo medication

Out of 13 trials included, there was no significant difference between the vitamin D cohort and placebo cohort in the majority of studies. Seven of the studies measured the development of diabetes as an outcome, comparing the onset of diabetes among participants who took vitamin D vs participants who took placebo medication. Out of these, six studies showed no statistically significant difference between onset of diabetes among each of the cohorts. Four studies provided a hazard ratio with all of them being 7 or greater. Figure 4 outlines the hazard ratios compiled. The longest lasting study was conducted by Jorde et al. and covered a span of five years. 50% of patients in the Vitamin D cohort developed diabetes, whereas 53.3% of patients in the placebo cohort developed diabetes, with a hazard ratio of 0.9. Pittas et al. yielded similar outcomes, with a study spanning four years and 24.4% of patients in the Vitamin D cohort developing diabetes compared to 26.9% of patients among the placebo cohort, with a hazard ratio of 0.88 [13]. There was however a significant difference in the vitamin D cohort and the placebo cohort seen in Dutta et al., with 10.9% of patients from the Vitamin D cohort developing diabetes vs 23.5% of patients from the placebo cohort developing diabetes. Additionally, in Ahmed et al., although incidence of diabetes was not recorded in the study, there was a significant rise in the OGIS index (an index of insulin sensitivity) seen in the Vitamin D cohort (15.3 ± 47.1 mL/min/m^2^) vs a significant decline in the OGIS index in the placebo cohort (-10.4 ± 44.7 mL/min/m^2^) - p=0.0029. Some individual studies reported in the level of impaired fasting glucose and HbA1c levels [6,7].

The effect of Vitamin D supplementation on insulin sensitivity was measured across the majority of the randomised controlled trials included. Table 3 includes the change in the Homeostatic Model Assessment for Insulin Resistance (HOMA-IR) from baseline in the cohorts that received vitamin D supplementation versus the cohorts that received placebo treatment.

**Table 3:** Change in HOMA-IR among Vitamin D and Placebo Cohorts

Author	Trial period	Vitamin D dose	No. of patients taking Vit D at end of study (n, %)	No. of patients taking placebo at end of study (n, %)	Change in HOMA-IR Among Vit D Cohort	Change in HOMA-IR Among Placebo Cohort	P value
Pittas et al. [11]	48 months	Vit D3 4000 IU/day	1201, 50%	1199, 50%	Not calculated	Not calculated	N/A
Dutta et al. [12]	28 months	Vit D3 60,000 IU/week for 8 weeks then monthly - Calcium supplemented in all groups	55, 52.8%	49, 47.2%	-0.5	-0.32	0.309
Jorde et al. [13]	60 months	Vit D3 20,000 IU/week	206, 49.5%	210, 51.5%	+0.46	+0.95	Not given
Salehpour et al. [14]	12 weeks	Vit D3 1000 IU/day	39, 50.6%	38, 49.4%	-5.7	-5.0	0.7
Al Thani et al. [15]	6 months	Vit D3 30,000 IU/week	57, 43.2%	75, 56.8%	-0.05	+0.06	0.45
Moreira-Lucas et al. [16]	24 weeks	Vit D3 28,000 IU/week via cheese	32, 50.8%	31, 49.2%	-1.04	-0.05	0.58
Oosterwerff et al. [17]	16 weeks	Vit D3 1200 IU/day (Calcium supplemented in both groups)	53, 48.2%	57, 51.8%	+0.1	0.0	Not given
Rasouli et al. [18]	24 months	Vit D3 4000 IU/day	888, 50%	886, 50%	+3.2	+0.9	0.07
Wallace et al. [19]	26 weeks	Vit D3 3000 IU/day	34, 53.1%	30, 46.9%	-1.4	-1.45	0.49
Forouhi et al. [20]	4 months	Vit D3 100,000 IU/month	99, 34.7%	92, 32.3%	Not calculated	Not calculated	N/A
		Vit D2 100,000 IU/month	94, 33%		Not calculated		N/A
Ahmed et al. [21]	12 weeks	Vit D3 60,000 IU/week	52, 51.4%	49, 48.6%	-0.2	+0.1	0.091
Kawahara et al. [22]	36 months	Eldecalcitol 30 IU/day	556, 50%	555, 50%	-0.2	+0.5	<0.001
Sollid et al. [23]	12 months	Vit D3 20,000 IU/week	242, 50%	242, 50%	+0.63	+0.76	Not given

Across all 13 trials there was a significant degree of heterogeneity. This was in terms of the dosages of Vitamin D, primary and secondary outcomes, research methodologies, duration of each study as well as the demographics of patients (gender, ethnicity, age range). Overall, 7,658 patients were included across all of the trials. Out of the six trials that included incidence of diabetes as a research outcome, 3,201 patients were in Vitamin D cohorts and 3,230 were in placebo cohorts. Out of the Vitamin D cohort, 20% of patients developed diabetes whereas out of the placebo cohort 23.3% of patients developed diabetes. This is outlined in Table 4.

**Table 4:** Pooled data across the studies showing the total number of patients in each cohort taken from the six studies that outline the incidence of diabetes as research outcome.

Summary of pooled data from included studies	
Total patients (n)	7658
Sex (n)%	Male – 52.6%
	Female 47.4%
Total number of patients taking Vitamin D (n)	3201
Total occurrence of Diabetes among Vitamin D Cohort (n)%	640 (20%)
Total number of patients taking Placebo (n)	3230
Total occurrence of Diabetes among Placebo cohort (n)%	751 (23.3%)

**Figure 5:**
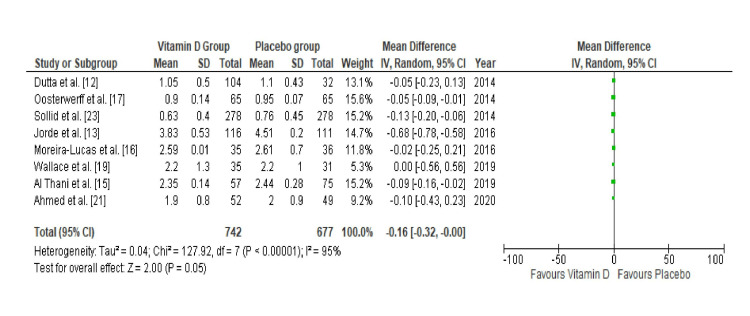
Meta-analysis from the included studies showing no obvious advantage of Vitamin D therapy over placebo

Meta analysis from the included studies did not show any advantage of the vitamin D therapy over placebo. We used random effect model due to variation in the studies included (Figure 5). There was significant heterogeneity present in the studies as show by I2 value of 95% hence random effect model was used.

Discussion

Multiple studies show some association between vitamin D status, its effect on blood glucose and the incidence of diabetes. As seen in the randomized controlled trials analyzed in this study, there is no conclusive evidence that supplementation with Vitamin D leads to a decrease in the incidence of Type 2 Diabetes. However, there does seem to be a correlation between Vitamin D levels and the degree of Insulin sensitivity in some of the trials, as seen by the influence of Vitamin D supplementation on patients’ HOMA-IR levels.

Larger studies had similar findings. The Melbourne Collaborative Cohort Study measured vitamin D levels in 628 participants who developed diabetes and 1,884 cohort subjects with a 4- to 11-year follow-up. It found that the lower the vitamin D levels, the higher the risk of developing T2DM and for each 25 nmol/L rise in 25(OH)D was associated with a 24% reduced risk of developing T2DM [24]. In another study by Pittas et al. on multiple observational studies and clinical trials, it found that three large trials had comparable risk reduction [25]. It showed around 12% reduction of developing diabetes in participants with prediabetes without vitamin D deficiency. Further subgroup analysis in one of the studies (D2d study) discovered that there was about 62% reduction in developing diabetes in those with low vitamin D status (less than 12 ng/mL) who took vitamin D supplements versus placebo. In addition, an estimated 48% converted to euglycemia. Moreover, a comprehensive analysis by He et al. weighed the effect of vitamin D in multiple randomized control trials (RCT) [26]. In 21 studies, pre-diabetic subjects with BMI less than 25 who had vitamin D supplementation had lower fasting plasma glucose (FPG) levels compared to placebo and vitamin D was most effective in decreasing FPG in subjects with 25(OH)D levels between 20 and 30 ng/mL. Thirteen studies assessed revealed that insulin sensitivity significantly improved in those subjects with a baseline 25(OH)D levels of greater than 30 ng/mL, via HOMA-IR. Significantly, in a subgroup analysis of six studies, there was a 16% reduction in the incidence of T2DM with vitamin D supplementation of greater than 2,000IU/day compared to controls [24-26].

Although the compelling evidence report a plausible relationship between Vitamin D status and the risk of developing T2DM, there are also those that conclude otherwise. In postmenopausal women with diverse racial backgrounds who had vitamin D deficiency that received vitamin D, about 6.3% later developed T2DM after an average 7.3-year surveillance. In this study, there was a lack of association of vitamin D levels and the incidence of T2DM after adjusting for multiple risk factors [27]. In another study by Qi et al., wherein 138 of the 596 subjects developed T2DM, showed that there was no statistical significance in vitamin D concentrations between those that developed T2DM versus those that did not develop the disease. Despite adjusting for confounders, the findings were consistent [28]. Lastly, going further via genetic analysis found that there was no association between a genetic predisposition from an increase in the levels of 25(OH)D and the risk of developing T2DM in a cohort of European descent. There was no genetic correlation of vitamin D with T2DM or other glycemic measures [29].

Evaluating the impact of the effect of vitamin D on different blood glucose parameters in vitamin D deficient subjects with various backgrounds revealed consistent results. In obese or overweight prediabetic individuals with vitamin D deficiency (< 50 nmol/L), the effect of vitamin D intake in glycemic measures versus placebo was minimal. The between-group change was 0.2 mmol/L, 0.4 mmol/mol and 0.2 for FPG, HbA1c and HOMA-IR, respectively [19]. Similarly, in non-western participants with high BMI, prediabetes and vitamin D deficiency in the Netherlands, supplementation of a moderate dose of vitamin D after four months had no notable change in impaired fasting glucose levels and impaired glucose tolerance versus placebo. HOMA-IR and HbA1c post intervention had comparable results despite adjusting for age, sex and BMI [17]. Comparable results were demonstrated in pre-diabetic Qatari subjects with vitamin D levels of less than 30 ng/mL. The vitamin D group had a mean change of 1.15 versus 1.09 (mmol/L) of the placebo group measured via the two-hour glucose tolerance test post six months intervention [15]. In another small-scale double-blind RCT with a 24-week follow-up, it found no notable difference in the primary outcome of the change in two-hour 75g oral glucose tolerance test between the vitamin D and placebo groups who were prediabetic with mostly 25(OH)D < 50 nmol/L, post vitamin D intervention [16]. Even secondary outcomes had no notable significance in the latter two studies. Also, for subjects with vitamin D deficiency of a proportion of about 50% to 58% with those below the 50 nmol/L threshold in both control and treatment groups with prediabetic risk, the change in HbA1c was insignificant, -0.05% for those given vitamin D2 and for vitamin D3 0.02% versus placebo [20].

Other studies with non-deficient subjects had conflicting findings. In healthy but overweight or obese women, after a 12-week vitamin D supplementation, there was mostly no significant difference seen in HOMA-IR, HbA1c and two-hour post prandial glucose from the vitamin D group versus placebo either biochemically or statistically. Even in terms of FPG, the placebo group had lower glucose levels than the vitamin D group. However, the mean FPG and HBa1c levels decreased in both groups and there was a correlation between Hba1c and vitamin D concentrations [14]. Likewise, in prediabetic subjects with high BMI, there was only a minimal change in β-cell function and insulin sensitivity in when compared between groups and their baseline. The average difference was 3.7% less in the vitamin D group versus 2.2% less in the placebo group after 24 months [18]. However, there was 4.9% change in seen in a subset of those with vitamin D of < 12ng/mL in the control group versus -13.6% in placebo.

Finally, two meta-analyses weighed the effect of vitamin D. Haroon and colleagues analyzed multiple studies that dealt with the effect of vitamin D on glycemic measures in mostly T2DM individuals. Although in short-term studies, which were defined as a follow up of three months or less, found that vitamin D had a beneficial effect on glycemic control, the evidence was inadequate due to validity of the studies. Further contradicting this finding were in long-term studies, defined as a follow-up of more than three months, which found no notable change in HbA1c or HOMA-IR [30]. Finally, Lips et al. evaluated majority of the vitamin D studies prior to 2016 - cohorts, RCTs and meta-analysis on healthy subjects, prediabetes and T2DM considering the variance of vitamin D levels in these subjects [31]. Since multiple studies were included, this covered various backgrounds such as ethnicity, obesity and gender. It found inconsistent results. Few studies exhibited a clinical benefit whereas others had no advantage over placebo. However, if there were any notable effect, they were often minute, challenging the clinical relevance of vitamin D in these studies [26]. For example, in over 15 RCTs, vitamin D had no effect in FPG, HbA1c or insulin resistance but in four trials in subjects with prediabetes, FPG decreased by 0.32 mmol/L.

In this systematic review, the degree of impact on vitamin D supplementation on patients’ incidence of Type 2 Diabetes, as well as on their Insulin sensitivity, varied from study to study. A significant degree of heterogeneity was noticed between the 13 trials included, in terms of type of Vitamin D supplementation used, primary and secondary outcomes, characteristics of participants included and duration of studies. Overall, it is more likely that Vitamin D does not delay the onset of Type 2 Diabetes. It is possible that there is a correlation between healthier patients having adequate Vitamin D levels, due to an increased level of exercise, healthier diets and increased sunlight exposure, who also have less insulin resistance compared to less healthy people who have more sedentary lifestyles, unhealthy diets, less sunlight exposure and lower Vitamin D levels.

Limitations

There was a variation in the study data between the trials ranging from sample size to different doses of supplemental Vitamin D, ethnicity of participants and baseline Vitamin D level. Additionally, factors that influence Vitamin D levels, such as sunlight exposure, level of physical activity and renal function were not controlled for in most trials. Similarly, factors that influence insulin resistance, such as diet, level of physical activity and family history were also not considered in most clinical trials. While identifying the impact of vitamin D supplementation on incidence of Type 2 Diabetes was the main objective of this systematic review, six of the thirteen randomized controlled trials identified did not include incidence of Type 2 Diabetes as a primary or secondary outcome. Among the trials that did include incidence of Type 2 Diabetes as a primary or secondary outcome, only four trials calculated hazard ratios. While HOMA-IR was used to identify the impact of Vitamin D supplementation on insulin resistance, two of the 13 trials did not calculate HOMA-IR and several did not calculate p-value.

## Conclusions

Overall, our systematic review yielded insufficient evidence to suggest that supplementation with Vitamin D delays the onset of T2DM significantly. However, supplementation with Vitamin D was seen to influence participants’ degree of Insulin resistance in a few trials. Whether this can result in the prevention of the development of T2DM will likely require randomized controlled trials. Further randomized controlled trials with matching doses of Vitamin D could help clarify the link between Vitamin D and Type 2 Diabetes in order to influence clinical guidelines on Vitamin D supplementation in patients with insulin resistance.
